# Analysis of microRNA-34a expression profile and rs2666433 variant in colorectal cancer: a pilot study

**DOI:** 10.1038/s41598-020-73951-y

**Published:** 2020-10-09

**Authors:** Manal S. Fawzy, Afaf T. Ibrahiem, Baraah T. Abu AlSel, Saleh A. Alghamdi, Eman A. Toraih

**Affiliations:** 1 Department of Biochemistry, Faculty of Medicine, Northern Border University, Arar, 1321 Saudi Arabia; 2 Department of Pathology, Faculty of Medicine, Northern Border University, Arar, 1321 Saudi Arabia; 3 Department of Microbiology, Faculty of Medicine, Northern Border University, Arar, 1321 Saudi Arabia; 4 Medical Genetics, Clinical Laboratory Department, College of Applied Medical Sciences, Taif University, Taif, 21944, Saudi Arabia; 5 Department of Medical Biochemistry and Molecular Biology, Faculty of Medicine, Suez Canal University, Ismailia, 41522 Egypt; 6 Department of Pathology, Faculty of Medicine, Mansoura University, Mansoura, 35516 Egypt; 7 Department of Surgery, School of Medicine, Tulane University, New Orleans, LA 70112 USA; 8 Genetics Unit, Department of Histology and Cell Biology, Faculty of Medicine, Suez Canal University, Ismailia, 41522 Egypt

**Keywords:** Molecular biology, Non-coding RNAs, miRNAs

## Abstract

MicroRNAs (miRNAs) are implicated in every stage of carcinogenesis and play an essential role as genetic biomarkers of cancer. We aimed to evaluate microRNA-34a gene (MIR34A) expression in colorectal cancer (CRC) tissues compared with non-cancer one and to preliminarily explore the association of one related variant to CRC risk. A total of 116 paraffin-embedded colon specimens were enrolled. MiR-34a was quantified by qPCR, and rs2666433 (A/G) genotyping was performed by TaqMan Real-Time PCR. Also, the somatic mutation burden was assessed. MIR34A expression in the CRC specimens was significantly upregulated (median = 21.50, IQR: 7.0–209.2; *P* = 0.001) relative to the non-cancer tissues. Allele (A) was highly prevalent in CRC tissues represented 0.56 (*P* < 0.001). AA/AG genotype carriers were 5.7 and 2.8 more likely to develop cancer than GG carriers. Tumor-normal tissue paired analysis revealed genotype concordance in 33 out of 58 tissue samples. Approximately 43% of the specimens showed a tendency for G to A shift. Additionally, a higher frequency of somatic mutation (92%) was observed in adenocarcinoma (*P* = 0.006). MIR34A expression and gene variant did not show associations with the clinicopathological data. However, G > A somatic mutation carriers had more prolonged DFS and OS. Bioinformatics analysis revealed miR-34a could target 30 genes that are implied in all steps of CRC tumorigenesis. In conclusion, this study confirms MIR34A upregulation in CRC tissues, and its rs2666433 (A/G) variant showed association with CRC and a high somatic mutation rate in cancer tissues. MiR-34a could provide a novel targeted therapy after validation in large-scale studies.

## Introduction

Colorectal cancer (CRC) represents the third most frequently diagnosed cancer, and the fourth prime cause of cancer-related mortality worldwide^1,2^. Although the etiology of colorectal cancer is multifactorial, genetic and epigenetic alterations of proto-oncogenes and tumor suppressor genes remain the fundamental mechanism of tumorigenesis^3^. Increasing interest in non-coding genomic sequences has revealed the recent implication of several classes of non-coding RNAs (ncRNAs), including microRNAs (miRNAs) as a type of posttranscriptional regulators in carcinogenesis^4^. This group of ncRNAs could exhibit differential expression in many types of cancer, including CRC^5^, and their dysregulation could promote every stage of carcinogenesis, including cell proliferation, invasion, and metastasis, and confer resistance to apoptosis through interaction with several intracellular signaling networks^6–8^. The thermodynamics of miRNA-mRNA target interactions may be influenced by single nucleotide polymorphisms (SNPs) occurring in the mature sequence of miRNA, resulting in target gene dysregulation with consequent phenotype variations and/or cancer susceptibility^9,10^.

MiR-34a is one of the emerging microRNAs that are implicated in many cancers, including CRC^11–14^. It has been shown that the expression of miR-34a is reduced in primary CRC tissues^15^. Moreover, CpG methylation of the miR-34a gene (MIR34A) promoter is detected in some colon cancer cell lines^16^, and its expression could be induced upon p53 activation^17^. Also, miR-34a could lower cell cycle progression through p53-dependent induction of p21 to alter colon cancer cell proliferation through direct or indirect regulation of the E2F transcription factor family^15^. Contrary to evidence on the pro-apoptotic role of miR-34a, however, also exists in the literature. It has been demonstrated that miR-34a may cooperate with p21 and 14-3-3σ to override the apoptotic signals generated by p53 activation^16^. As a controversy of miR-34a role in CRC still exists and also as sequence variations in the miRNA-binding sites could affect either the expression level and/or the oncogenic or tumor-suppressing functions of cancer-associated miRNAs^6,9,10^, the current study aims to analyze MIR34A expression and rs2666433 (A/G) variant in preliminary samples of archived CRC tissue specimens in comparison to non-cancer tissues and correlate the results to the available clinicopathological data. This could help improve our understanding of the impact of such type of miRNAs in CRC and its potential role as a candidate for the future molecular-based individualized therapy of such lethal cancer.

## Results

### In silico analysis of miR-34a

**Figure 1:**
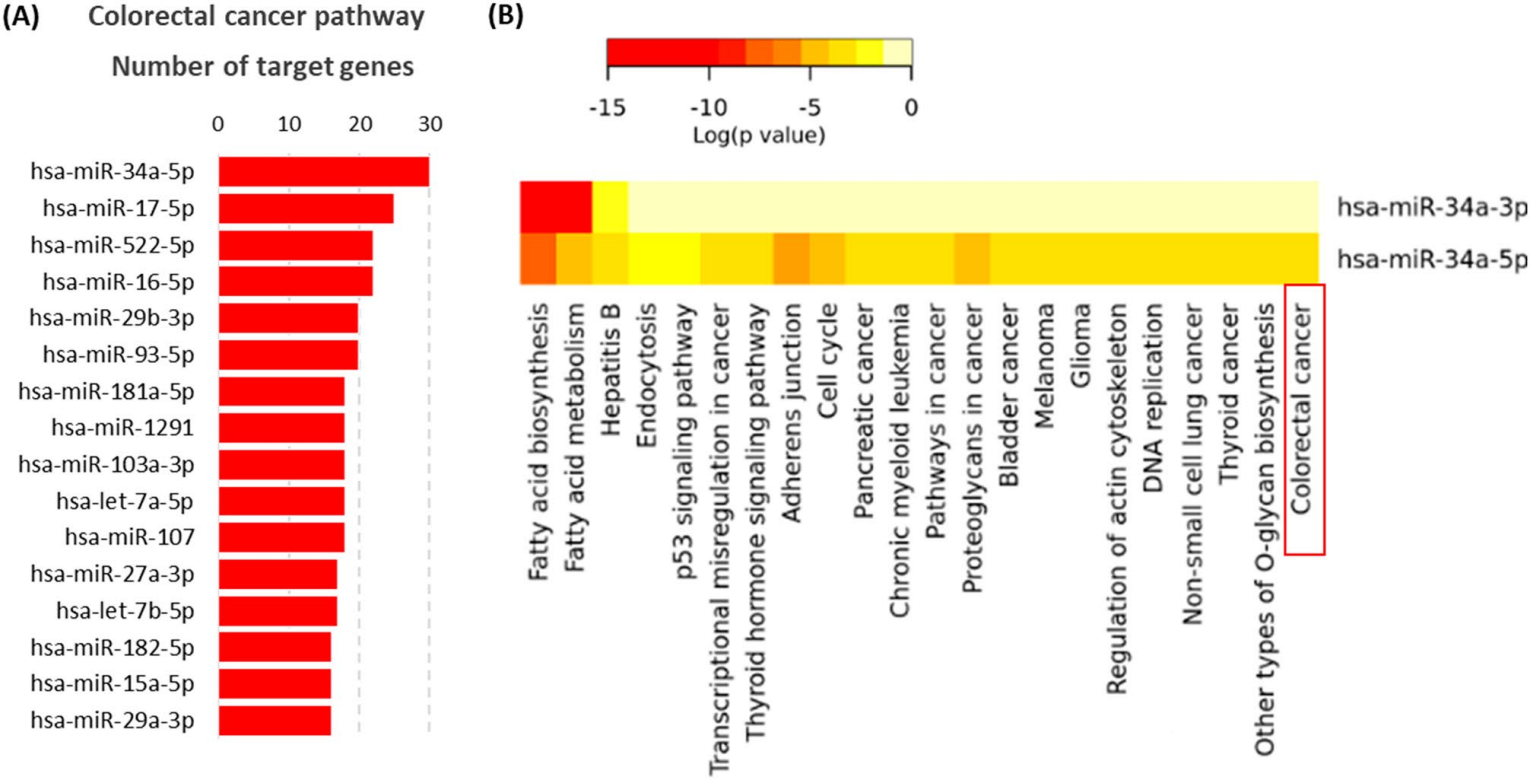
Top microRNAs involved in the colorectal cancer pathway. (**A**) microRNAs involved in the colorectal cancer KEGG pathway (https://www.kegg.jp/kegg/kegg1.html)^18,19^. (**B**) Functional enrichment pathway analysis of miR-34a target genes. [Data source: Diana lab tools]. The top bar indicates the log (P-value) of the implication of the specified pathway in colon cancer. The direction towards red color indicates more significance as the log P < 0.05 equivalent to P < -1.30.

**Figure 2:**
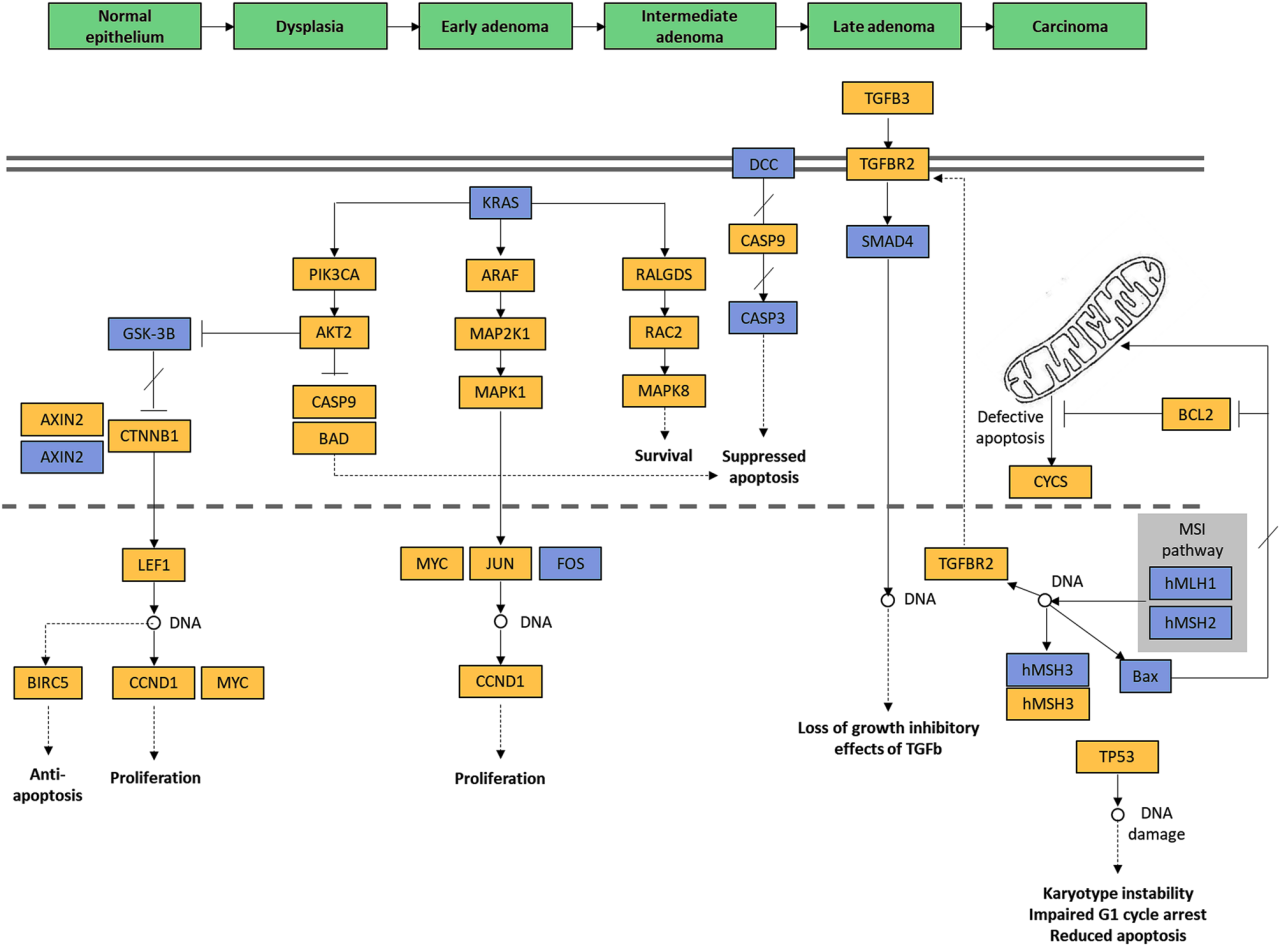
MicroRNA-34a targets the colorectal cancer pathway. Genes targeted by miRNA34a are colored in orange. In the CRC pathway (KEGG: hsa05210)^18,19^, miR-34a-5p significantly targets 30 genes (*P* = 0.0013); including B-Raf proto-oncogene, serine/threonine kinase (*BRAF*), Ras-Related C3 Botulinum Toxin Substrate 2 (*RAC2*), phosphoinositide-3-kinase regulatory subunit 2 (*PIK3R2*), phosphatidylinositol-4,5-bisphosphate 3-kinase catalytic subunit alpha (*PIK3CA*), RAF1, B-cell lymphoma-2 (*BCL2*), BCL2 associated agonist of cell death (*BAD*), Baculoviral IAP repeat containing 5 (*BIRC5*), transforming growth factor beta 1 (*TGFB1*), TGFB3, TGFB-receptor 2 (*TGFBR2*), TCF7L1 (Transcription Factor 7 Like 1), ARAF, Tumor Protein P53 (*TP53*), AKT serine/threonine kinase 2 (*AKT2*), Jun proto-oncogene, AP-1 transcription factor subunit (*JUN*), cyclin D1 (*CCND1*), mothers against DPP homolog 4 (*SMAD4*), catenin beta 1 (*CTNNB1*), MutS homolog 6 (*MSH6*), axis inhibition proteins 2 (*AXIN2*), MYC proto-oncogene, BHLH transcription factor (*MYC*), mitogen-activated protein kinase 1 (*MAPK1*), MAPK3, MAPK8, mitogen-activated protein kinase kinase 1 (*MAP2K1*), caspase-9 (*CASP9*), Ral guanine nucleotide dissociation stimulator (*RALGDS*), lymphoid enhancer binding factor 1 (*LEF1*), cytochrome C, somatic (*CYCS*).

Our bioinformatics analysis identified miR-34a-5p to be the most highly significant non-coding microRNA enriched in the colorectal cancer pathway (Fig. 1). It can complement and bind 30 target genes and fine-tuning their expression profile (Fig. 2).

### Functional enrichment analysis of miR-34a

Both miR-34a-5p and miR-34a-3p were mostly significantly involved in two pathways: namely fatty acid biosynthesis (hsa00061) and fatty acid metabolism (hsa01212). miR-34a-5p was also identified to target specific cancer types; including colorectal cancer, thyroid cancer, non-small cell lung cancer, chronic myeloid leukemia, bladder cancer, pancreatic cancer, glioma, and melanoma, in addition to multiple cancer-related pathways as cell cycle, pathways in cancer, p53 signaling pathway, and proteoglycans in cancer.

**Figure 3:**
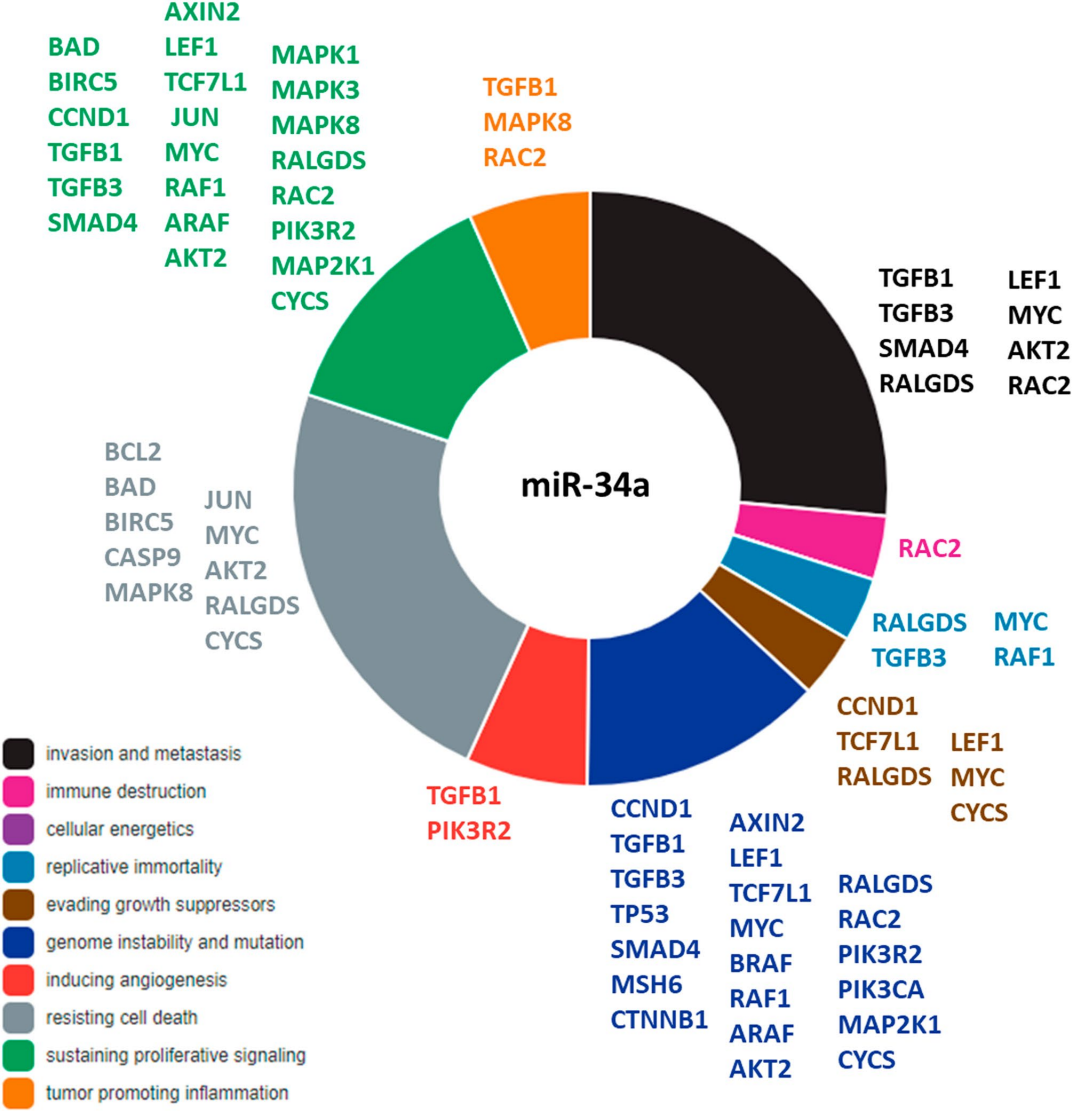
MicroRNA-34a is involved in the cancer hallmarks. The thirty target genes in the colorectal cancer pathway are functionally enriched in diverse cancer hallmarks. Data source: Cancer Hallmarks Analytics Tool (https://chat.lionproject.net/)20. Each color indicates a specific cancer hallmark, as indicated in the left colored legend.

In the CRC pathway (KEGG: hsa05210)^18,19^, miR-34a-5p significantly targets 30 genes (P = 0.0013); which are involved in all steps of colorectal development and progression (Fig. 2). These gene lists included apoptotic genes (*BCL2*, *BAD*, *BIRC5*, and *CASP9*), proliferative genes (*CCND1*, *TGFB1*, and *TGFB3*), tumor suppressor genes (*TGFBR2*, *TP53*, and *SMAD4*), DNA repair gene (*MSH6*), oncogene (*CTNNB1*), transcription factors (*JUN*, *MYC*, *TCF7L1*), and serine-threonine kinases (*BRAF*, *RAF1*, *ARAF*, *AKT2*, *MAPK1*, *MAPK3*, and *MAPK8*) (Fig. 2). Enrichment of miR-34a in hallmarks of cancer^20^ revealed to be involved in two main functions: namely resisting cell death (gray color, Fig. 3) and tumor invasion and metastasis (black color, Fig. 3).

### Genotyping of MIR34A variant

Genotype frequencies of rs2666433 agreed with HWE in patients (P = 0.34) and controls (P = 0.13). MAF (A allele) accounted for 0.31 in controls. According to the 1000 Genome Project, the same allele frequencies were 0.43 in Africans, 0.31 in East Asians, 0.23 in South Asians, 0.17 in Americans, and 0.09 in Europeans.

### Impact of genotypes on cancer risk

On the comparison between malignant and adjacent colon tissues, A allele was highly prevalent in cancer tissues representing a frequency of 0.56, P < 0.001. Correspondingly, AA and AG genotypes were predominant in cancer specimens (34.5% and 43.1%) compared to counterpart non-cancerous tissues (13.8% and 34.5%), respectively. AA and AG were 5.7 and 2.8 more likely to develop cancer than GG (AA versus GG: OR 5.76, 95%CI: 2.02–16.43, AG versus GG: OR 2.88, 95% CI 1.20–6.93) (Table 1).

**Table 1:** Genetic association models for MIR34A variant and cancer risk.

Genetic model	Genotype	Control	Cancer	OR (95% CI)	*P-*value
Heterozygote comparison	GG	30 (51.7)	13 (22.4)	*Reference*	**0.017**
	AG	20 (34.5)	25 (43.1)	2.88 (1.20–6.93)	
Homozygote comparison	GG	30 (51.7)	13 (22.4)	*Reference*	**0.001**
	AA	8 (13.8)	20 (34.45)	5.76 (2.02–16.43)	
Dominant model	GG	50 (86.2)	38 (65.5)	*Reference*	**0.001**
	AA + AG	28 (13.8)	45 (77.6)	3.71 (1.66–8.29)	
Recessive model	GG + AG	30 (51.7)	13 (22.4)	*Reference*	**0.008**
	AA	8 (13.8)	20 (34.45)	3.29 (1.31–8.27)	
Over-dominant model	GG + AA	38 (65.5)	33 (56.9)	*Reference*	0.34
	AG	20 (34.5)	25 (43.1)	1.44 (0.68–3.05)	
Allelic model	G	80 (69.0)	51 (44.0)	*Reference*	** < 0.001**
	A	36 (31.0)	65 (56.0)	2.83 (1.65–4.84)	

Values are shown as numbers (%). A Chi-square test was used. OR (95% CI), odds ratio, and 95% confidence interval. Bold values indicate statistically significant at *P* < 0.05.

### Somatic mutation burden analysis

Tumor-normal paired analysis revealed genotype concordance in 33 out of 58 tissue samples. However, the rest of the specimen (43.1%) showed a tendency for G to A shift; 8 (13.8%) controls with AG genotype were substituted to AA in paired adjacent cancer tissue, 13 non-malignant samples (22.4%) changed from GG to AG, and 4 samples (6.9%) with GG genotype showed double mutations to AA at both gene loci in malignant tissues derived from the same patients (Table 2).

**Table 2:** Somatic mutations of rs2666433 (A/G) genotypes in cancer and paired non-cancer tissues.

	Cancer			Total	*P*-value
	AA	AG	GG		
**Control**					
AA	8 (13.8)	0 (0.0)	0 (0.0)	8 (13.8)	**0.021**
AG	8 (13.8)	12 (20.7)	0 (0.0)	20 (34.5)	
GG	4 (6.9)	13 (22.4)	13 (22.4)	30 (51.7)	
Total	20 (34.5)	25 (43.1)	13 (22.4)	58 (100)	

Values are shown as numbers (% from total participants). McNemar's test was used. The bold value indicates statistically significant at *P* < 0.05.

### Transcriptomic signature of miR-34a in CRC

**Figure 4:**
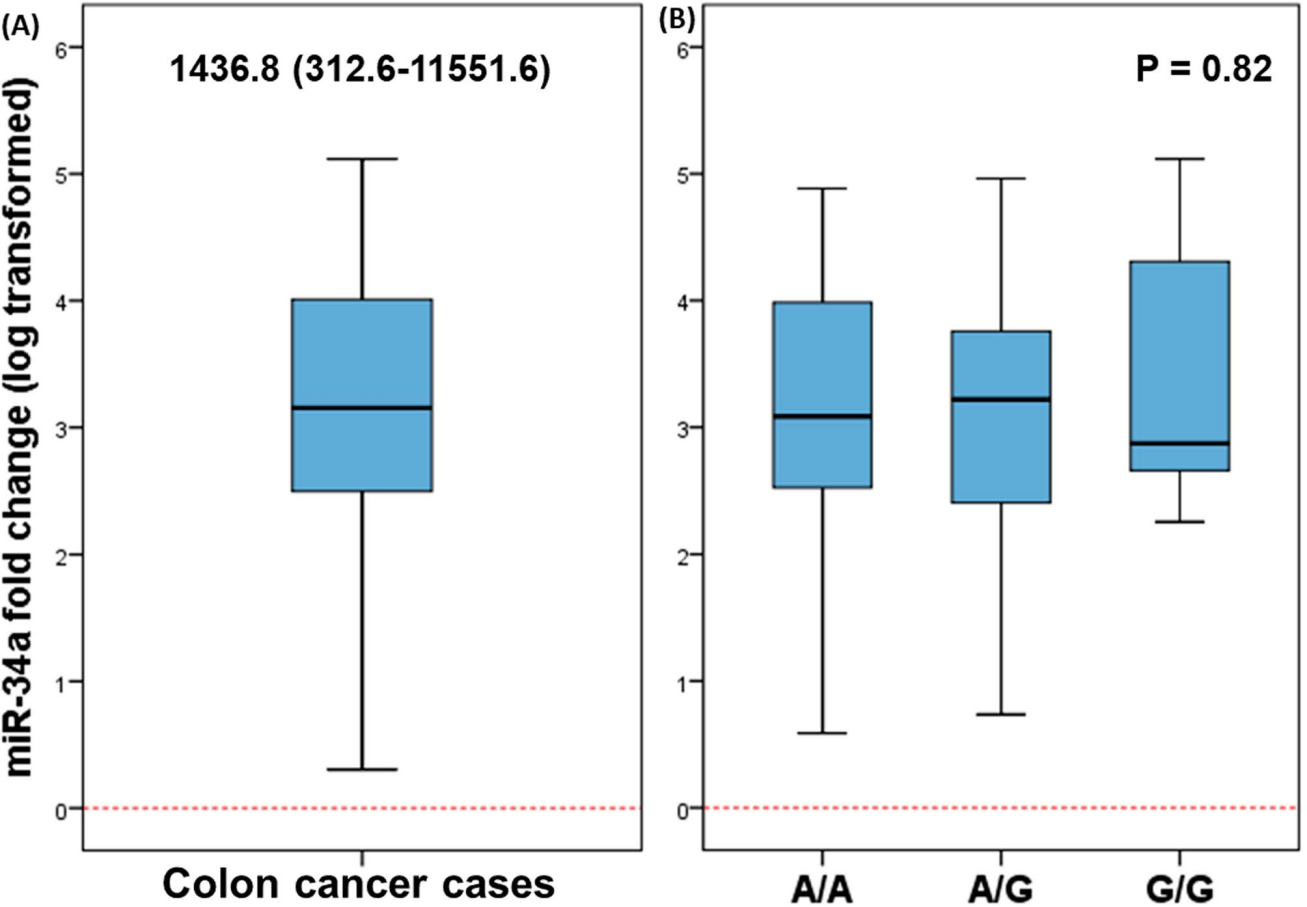
The relative expression profile of the MIR34A gene in colon cancer specimens. Data are shown as medians and quartiles. Box plot values were log-transformed as data were non-parametric. The box defines upper and lower quartiles (25 and 75%, respectively), and the error bars indicate upper and lower adjacent limits. The fold change was normalized to RNU6B and calculated using the delta-delta quantitative cycle (Cq) method [= 2^(-DDCq)^] in comparison to non-cancer adjacent tissues. The red dotted line represents the control level. Mann–Whitney U and Kruskal–Wallis tests were applied. (**A**) Overall samples. (**B**) Stratified by rs2666433 genotype.

The mean quantitative threshold cycle (Cq) for RNU6B was 38.5 ± 3.23 in cancer specimens compared to 39.4 ± 5.38 in paired samples (P > 0.05). Hence, RNU6B was used in the downstream analysis as an endogenous control. In contrast, Cq of miR-34a-5p varied significantly between the study population; 35.1 ± 2.6 in cancer and 36.7 ± 3.8 in non-cancer (P < 0.001). Marked over-expression of miR-34a was observed in the cancer specimen with a median and quartile levels of 1436.8 (312.6–11,551.6) (Fig. 4A). Therefore, log-transformed values were used for further analyses. No differential expression level of miR-34a was found in patients with different rs2666433 genotypes (P = 0.82) (Fig. 4B).

### Association of MIR34A expression and variant with clinicopathological features

As depicted in Table 3 and Suppl. Fig. S1, no association was found between any of the patient characteristics and miR-34a expression or polymorphism. However, patients harboring G > A somatic mutation had a more prolonged DFS (P = 0.003) and OS (P < 0.001) than non-carriers. Also, unlike all other types of colon cancer, a higher frequency of somatic mutation (92%) was observed in adenocarcinoma (P = 0.006) (Table 4).

**Table 3:** Univariate association analysis of MIR34A expression and variant with clinicopathological features.

Characteristics	No. of cases	Fold change	*P-*value	Genotype			*P-*value
		Mean		AA	AG	GG	
**Age (years)**							
≤ 55	27 (46.6)	3.14 ± 1.08	0.857	10 (50.0)	12 (48.0)	5 (38.5)	0.795
> 55	31 (53.4)	3.25 ± 1.15		10 (50.0)	13 (52.0)	8 (61.5)	
**Sex**							
Female	22 (37.9)	3.18 ± 1.22	0.916	8 (40.0)	9 (36.0)	5 (38.5)	0.962
Male	36 (62.1)	3.21 ± 1.05		12 (60.0)	16 (6.0)	8 (61.5)	
**Location**							
Ascending	26 (44.8)	3.03 ± 1.00	0.204	11 (55.0)	9 (3.0)	6 (46.2)	0.797
Transverse	4 (6.9)	3.10 ± 0.92		1 (5.0)	(8.0)	1 (7.7)	
Descending	28 (48.3)	3.37 ± 1.23		8 (40.0)	14 (56.)	6 (46.2)	
**Type**							
Adeno	39 (67.2)	3.14 ± 1.13	0.488	17 (85.0)	16 (64.)	6 (46.2)	0.162
Muci	8 (13.8)	3.63 ± 0.97		1 (5.0)	5 (20.0)	2 (15.4)	
Signet	6 (10.3)	3.16 ± 0.64		0 (0.0)	3 (12.0)	3 (23.1)	
Undif	5 (8.60	2.98 ± 1.66		2 (10.0)	1 (4.0)	2 (15.4)	
**Grade**							
G1	8 (13.8)	3.56 ± 1.10	0.401	3 (15.0)	4 (16.0)	1 (7.7)	0.390
G2	32 (55.2)	3.14 ± 1.15		12 (60.0)	15 (60.0)	5 (38.5)	
G3	18 (31.0)	3.14 ± 1.06		5 (25.0)	6 (24.0)	7 (53.8)	
**Tumor size**							
T1	5 (8.6)	3.70 ± 0.95	0.874	3 (15.0)	2 (8.0)	0 (0.0)	0.518
T2	28 (48.3)	3.12 ± 1.18		10 (50.0)	10 (40.0)	8 (61.5)	
T3	15 (25.9)	3.25 ± 1.13		3 (15.0)	8 (32.0)	4 (30.8)	
T4	10 (17.2)	3.09 ± 1.00		4 (20.0)	5 (20.0)	1 (7.7)	
**LN invasion**							
N0	25 (43.1)	3.36 ± 1.10	0.974	9 (45.0)	10 (40.0)	6 (46.2)	0.761
N1	22 (379)	3.29 ± 0.95		9 (45.0)	9 (36.0)	4 (30.8)	
N2	11 (19.0)	2.67 ± 1.35		2 (10.0)	6 (24.0)	3 (23.1)	
**Metastasis**							
M0	47 (81.0)	3.23 ± 1.09	0.545	17 (85.0)	19 (76.0)	11 (84.6)	0.696
M1	11 (19.0)	3.07 ± 1.24		3 (15.0)	6 (24.00	2 (15.4)	
**LVI**							
No	36 (62.1)	3.12 ± 1.15	0.974	13 (65.0)	17 (68.0)	6 (46.2)	0.398
Yes	22 (37.9)	3.33 ± 1.06		7 (35.0)	8 (32.0)	7 (53.8)	
Dukes*							
A	14 (24.1)	3.49 ± 1.21	0.361	6 (30.0)	4 (16.0)	4 (30.8)	0.856
B	9 (15.5)	3.34 ± 0.94		2 (10.0)	5 (20.0)	2 (15.4)	
C	24 (41.4)	3.04 ± 1.07		9 (45.)	10 (40.0)	5 (38.5)	
D	11 (19.0)	3.07 ± 1.24		3 (15.0)	6 (24.0)	2 (15.4)	
**Relapse**							
No	37 (64.9)	3.34 ± 1.01	0.803	15 (75.0)	12 (50.0)	10 (76.9)	0.131
Yes	20 (35.1)	2.96 ± 1.28		5 (25.0)	12 (50.0)	3 (23.1)	
**Died**							
No	18 (32.1)	3.18 ± 1.15	0.682	3 (15.0)	9 (39.1)	6 (46.2)	0.112
Yes	38 (67.9)	3.22 ± 1.13		17 (85.0)	14 (60.9)	7 (53.8)	
**DFS (months)**							
Mean	58 (100)	43.0 ± 11.8		45.40 ± 9.27	43.48 ± 14.36	38.46 ± 9.40	0.256
**OS (months)**							
Mean	58 (100)	47.7 ± 11.6		49.25 ± 9.37	48.96 ± 14.57	43.15 ± 7.46	0.276

Data are shown as mean ± standard deviation or frequency as number (percentage). Chi-square, student's t, ANOVA tests were used.

*Adeno* adenocarcinoma; *Muci* Mucinous carcinoma; *Signet* Signet ring carcinoma; *Undif* Undifferentiated carcinoma; *LN* lymph node; *LVI* lymphovascular invasion; *DFS* disease-free survival; *OS* overall survival.

*Tumors were staged clinically according to the TNM classification.

**Table 4:** Comparison between patients with and without somatic mutation.

Number Mean	Without G > A mutation	With G > A mutation	*P*-value
**Age (years)**			
≤ 55	12 (36.4)	15 (60.0)	0.111
> 55	21 (63.6)	10 (40.0)	
**Sex**			
F	10 (30.3)	12 (48.0)	0.186
M	23 (69.7)	13 (52.0)	
**Location**			
Ascending	17 (51.5)	9 (36.0)	0.500
Transverse	2 (6.06)	2 (8.0)	
Descending	14 (42.4)	14 (56.0)	
**Type**			
Adeno	16 (48.4)	23 (92.0)	**0.006**
Muci	7 (21.2)	1 (4.0)	
Signet	5 (15.1)	1 (4.0)	
Undif	5 (15.1)	0 (0.0)	
**Grade**			
G1	4 (12.1)	4 (16.0)	0.097
G2	15 (45.4)	17 (68.0)	
G3	14 (42.4)	4 (16.0)	
**Tumor size**			
T1	3 (9.09)	2 (8.0)	0.622
T2	16 (48.4)	12 (48.0)	
T3	10 (30.3)	5 (20.0)	
T4	4 (12.12)	6 (24.0)	
**LN invasion**			
N0	14 (42.42)	11 (44.0)	0.877
N1	12 (36.36)	10 (40.0)	
N2	7 (21.21)	4 (16.0)	
**Metastasis**			
M0	27 (81.82)	20 (80.0	0.861
M1	6 (18.18)	5 (20.0)	
**LVI**			
No	21 (63.64)	15 (60.0)	0.792
Yes	12 (36.36)	10 (40.0)	
Dukes*			
A	10 (30.3)	4 (16.0)	0.599
B	4 (12.12)	5 (20.0)	
C	13 (39.39)	11 (44.0)	
D	6 (18.18)	5 (20.0)	
**Relapse**			
No	22 (68.75)	15 (60.0)	0.580
Yes	10 (31.25)	10 (4000)	
**Died**			
No	12 (38.71)	6 (24.0)	0.267
Yes	19 (61.29)	19 (76.0)	
**DFS (months)**	39.12 ± 12.19	48.16 ± 9.40	**0.003**
≥ 48	9 (27.27)	18 (72.0)	**0.001**
< 48	24 (72.73)	7 (28.0)	
**OS (months)**	42.73 ± 11.51	54.40 ± 8.20	** < 0.001**
≥ 48	12 (36.36)	22 (88.0)	** < 0.001**
< 48	21 (63.64)	3 (12.0)	
MIR34A expression	3.23 ± 1.02	3.15 ± 1.23	0.788

Data are shown as mean ± standard deviation or frequency as number (percentage). Chi-square, student's t, ANOVA tests were used.

*Adeno* adenocarcinoma; *Muci* Mucinous carcinoma; *Signet* Signet ring carcinoma; *Undif* undifferentiated carcinoma; *LN* lymph node; *LVI* lymphovascular invasion; *DFS* disease-free survival; *OS* overall survival.

*Tumors were staged clinically according to the TNM classification of colon cancer. Bold values indicate statistically significant at *P* < 0.05.

## Discussion

Given the advancement in the "high-throughput genome-wide profiling" and "screening technologies", newly emerged miRNA signatures and several "miRNA–mRNA" crosstalk have been identified in CRC^21^. An example of those signatures is the MIR34A gene expression, which plays a critical role in all stages of colorectal carcinogenesis, starting from colon epithelium proliferation, dysplasia, early/late adenoma, and progression to malignant neoplasm (Fig. 2). The present study identified significant upregulation of miR-34a in CRC tissues relative to normal tissues. In contrast to other studies that reported p53- and other molecular players-mediated miR-34a down-regulation in CRC tissue/plasma samples^15,22–27^, our finding was in line with that of Aherne and colleagues, who found a significant increase of miR-34a tissue expression in early-stage CRC samples compared to non-malignant ones and in colorectal adenomas relative to polyp and normal tissues^28^. Interestingly, the latter findings corresponded to the same changes in miR-34a circulating levels in CRC patients, in an independent cohort explored by the same authors. Brunet et al. also, reported overexpression of miR-34a in CRC (stage III) tissue samples relative to normal ones, which support the oncogenic role of miR-34a in the CRC. The observed controversy in results̕ reproducibility in aforementioned studies could reflect variable miRNA expression signatures due to disparities in participant age and/or time of sample collection^27^, varied sex distribution in the specified study^29^, racial difference^30^, tumor sample heterogeneity, and different detection approaches^28^. Additionally, miR-34a has multiple targets even in the same type of cancer^31^ (Figs. 2 and 3), as well as being itself a target for other coding^32,33^ and non-coding RNAs^34–38^, creating multiple circRNAs/lncRNA-miRNA-mRNA crosstalk networks that either promote or inhibit carcinogenesis in a spatial-, temporal- and cell type-specific pattern. In this sense, more "gene–gene interaction" analyses will better uncover miR-34a and its regulatory genes implicated in the pathogenesis of CRC.

Several molecular pathways have been identified to mediate the miRNA-34a role in this context, including Notch-1 and Notch-2 pathway suppression, which implicated in self-renewal and colon stem cells differentiation^39,40^, tumor-initiating cells (cancer stem cells) regulation^41,42^, and Fos-related antigen-1 (FRA1) targeting^23^ which plays an essential role in mediating the crosstalk between the oncogenic RAS-ERK and TGFβ signaling networks implied in "epithelial-mesenchymal plasticity" during CRC progression^43^.

It has been reported that miRNA SNPs might also cause an aberrant function of the miRNA in regulating the putative target genes^44^. Previous researches have shown that MIR34A variants could modulate the susceptibility of individuals to multiple human cancers, including osteosarcoma^45^, colon cancer^46^, and breast cancer^47^. The present results revealed that MIR34A rs2666433 AA and AG genotype carriers were 5.7 and 2.8 more likely to develop cancer than GG carriers. A part of one recent publication related to the study of the MIR34A rs2666433 association with ischemic stroke in Chinese population^48^, the impact of this variant on CRC risk (or other types of cancer) has not been reported previously. Interestingly, we also found that nearly 43% of the cancer tissues showed a tendency for G to A shift, and a higher frequency of somatic mutation (92%) was observed in the adenocarcinoma subtype of CRC. Although the normal and cancer colon tissues were exposed to the same environmental insult, only the cancer tissues showed the transformation into malignancy, which confirms the contribution of the cell genetic and epigenetic makeup to this transformation. Recently, Sun et al., suggested that the rs2666433 variant may affect the binding of transcription factors to MIR34A promoter sequences^49^.

Furthermore, Wei et al. reported that ischemic stroke patients with rs2666433 (AA) genotype had a higher level of miR-34a than those with (GG + GA) genotypes^48^, suggesting that rs2666433 may influence miR-34a expression level in their population. However, we could not find a significant association between the specified microRNA variant and its tissue expression levels in the present samples. The authors confirm the specificity of miRNAs, which is related to the type of the disease (ischemic stroke vs. cancer), the type of cancer (the CRC in the present study), the type of samples (body fluids vs. tissues), and the study population (i.e. ethnicity) among others. The negative result could also be partly related to the limited sample size that warrants further large-scale studies to confirm this finding in CRC tissues. It's worth noting that although this limitation above, an essential element of the validity of our study is its agreement with HWE in both study groups, particularly the controls which exclude any genotyping errors or guided sample selection by the authors. Another raised limitation in this study could be related to evaluation of the study variant in FFPE normal colon tissue samples, which, however, is "a very common source for DNA extraction in the studies regarding microRNAs"^47^.

In conclusion, the present study revealed miR-34a upregulation in CRC tissues compared to paired non-cancer ones. Moreover, for the first time, the authors reported an association between MIR34A rs2666433 (A/G) variant and CRC risk in the study population with a high rate of the specified miRNA mutation in cancer tissues relative to controls. These results could support the previous evidence of miR-34a implication in CRC pathogenesis and its potential use as a biomarker with other molecular panels or as an individualized therapeutic target in the near future. For results validation, further large-scale studies, including several miRNAs combinations in ethnic different populations, are highly recommended.

## Methods

### Bioinformatics-based selection of microRNA and its variant

Screening microRNAs involved in the colorectal cancer pathway [KEGG: hsa05210]^18,19^, we found 621 miRNAs targeting this particular pathway, in which miR-34a-5p ranked the top among them (Fig. 1). Experimentally validated gene targets for miR-34a-5p and miR-34a-3p were obtained from DIANA (https://diana.imis.athena-innovation.gr/), miRDB (https://mirdb.org), miRTarBase v.20 (https://mirtarbase.mbc.nctu.edu.tw/), TargetScanHuman v6.2 (https://www.targetscan. org/), PicTar (https://pictar.mdc-berlin.de/), and miRNAMap v2.0. Network analysis of target genes was carried out via a network analyst, web server (https://www.networkanalyst.ca). Functional enrichment pathway analysis was performed using miRTar. Human tool (https://miRTar.mbc.nctu.edu.tw/) and DIANA-miRPath v3.0 (https://snf-515788.vm.okeanos.grnet.gr/index.php?r=mirpath/reverse) which integrate all miRNA targets at the coding sequences (CDS), 3′ untranslated region )3′UTR), or 5′UTR regions into significant Kyoto encyclopedia of genes and genomes (KEGG) pathways and gene ontology (GO). Next, miR-34a disease interactions were retrieved from the PhenomiR database (https://mips.helmholtz-muenchen.de/phenomir/), miR2Disease database (https://www.miR2Disease.org), miRCancer database (https://mircancer.ecu.edu/about.jsp), ExcellmiRDB (www.excellmirdb.brfjaisalmer.com/), and Human miRNA Disease Database (HMDD) v2.0 (https://cmbi.bjmu.edu.cn/hmdd).

The genetic variant of MIR34A (rs2666433; A>G) is located 2 kb upstream to the mature miRNA sequence and within the intron of the MIR34A host gene (MIR34AHG; n.333-1283T > C) (ensemble.org). It was registered with an overall minor allele frequency (MAF: A allele) in the 1000Genome Project of 0.260.

### Sample collection

A total of 116 formalin-fixed, paraffin-embedded (FFPE) specimens were collected retrospectively, including 58 CRC samples and paired 58 non-cancer colon tissues. Specimens were obtained from patients who underwent colon resection for histologically confirmed carcinoma. Paired controls were adjacent tissues obtained from the surgical free margins of each specimen and recorded to be normal by microscopic examination before its parafinization. All retrieved cases were archived in the Department of Pathology, Mansoura University, between 2013 and 2017. Patient data were obtained from medical records. There was no history of neoadjuvant therapy before surgery. Direct contact of patients was performed to complete missing data and follow-up (the last contact was in July 2019). The available follow-up period ranged from 20 to 68 months. Samples with incomplete clinical data or follow-up period, history of receiving any treatment before surgery, and/or diagnosis with malignant disease primarily arising from other organs were excluded. The study was conducted according to the ethical and legal guidelines adopted by the Declaration of Helsinki. Ethical approval for this study was granted by the local Research Ethics Committee (No. MED-2018-3-9-F-7825). The informed consent from the patients was waived from the ethical committee as the authors worked on archived samples.

### Histopathological examination

Specimens included adenocarcinoma (n = 39; 67.2%), mucinous carcinoma (n = 8; 13.8%), signet ring cell carcinoma (n = 6; 10.3%), and undifferentiated type (n = 5; 8.60%). Apart from the limited sample size in this pilot study, the low frequency of undifferentiated carcinoma subtype in our cases could be congruent with the relative low frequency of such type of CRC as evidenced previously^50^, and including only the confirmed immunohistochemical staining for cytokeratin (CK) cases which could additionally contribute to the low number of such cases. Sections were examined for histopathologic diagnosis and tumor, node, metastasis (TNM) staging by an expert pathologist^51^. Other sections (5 to 8 μm thick) for cancer and paired non-cancer tissues were collected in separate Eppendorf tubes for both miRNA expression and SNP identification analyses.

### Gene expression profiling

Total RNAwas purified from the FFPE colon sections using a Qiagen miRNeasy FFPE Kit (Cat # 217504) following the manufacturer's instructions^12^. RNAconcentration and purity were assessed by a NanoDrop ND-1000 spectrophotometer (NanoDrop Tech., Inc. Wilmington, DE, USA), and the integrity was checked by gel electrophoresis. Specific complementary DNA(cDNA) was prepared using TaqMan MicroRNA Reverse Transcription (RT) kit (P/N 4366596; (Thermo Fisher Scientific, Applied Biosystems, Foster City, CA, USA) for miR-34a-5p (assay ID 000426) as described in our previous publication^52^. RNU6B exhibited a uniform and stable expression in colon tissues with no significant difference between cancer and non-cancer samples; thus was used as an endogenous control (assay ID 001093). T-Professional Basic, Biometra PCRSystem (Biometra, Germany) was used. Appropriate negative controls were applied in each run to exclude amplicon contamination. The PCRreactions were carried out in triplicate in StepOne Real-Time PCRSystem (Applied Biosystems) using specific TaqMan small RNA assay^53^. All the steps of the quantitative Real-Time reverse transcription-polymerase chain reaction (qRT-PCR) were run according to the Minimum Information for Publication of Quantitative Real-Time PCR Experiments (MIQE) guidelines^54^. The relative MIR34A expression levels were calculated using the LIVAK method (2^-ΔΔ*C*q^), where Delta-Delta quantitative cycle (C_q_) = (*C*_*q*_ MIR34A − *C*_*q*_ RNU6B)_CRC_ − (*C*_*q*_ MIR34A − *C*_*q*_ RNU6B)_NAT_^55^.

### Allelic discrimination analysis

QIAamp DNA FFPE tissue kit (QIAGEN, 56,404) was used for DNA extraction according to the guided protocol. DNA quality/purity was evaluated, as mentioned above. DNA samples from the 58 cancer specimens and 58 non-cancer tissues were genotyped for the MIR34A variant (rs2666433 (A/G), assay ID C___2800266_10) using Taqman Real-Time PCR method as detailed previously^56^. Appropriate negative controls were applied in each PCR run to avoid the false positive of amplicon contamination. Real-time PCR amplification was performed on StepOne Real-Time PCR System (Applied Biosystems) using the following conditions: an initial hold (95 °C for 10 min) followed by a 40-cycle two-step PCR (95 °C denaturation for 15 s and annealing/extension 60 °C for 1 min). Allelic discrimination was called by the SDS software version 1.3.1 (Applied Biosystems). Genotyping was performed by two persons independently blinded to case/control status. Ten percent of the randomly selected samples were re-genotyped in separate runs to exclude the possibility of false genotype calls, with 100% concordance of the results.

### Statistical analysis

Data were managed using SPSS version 24.0, the R packages, and GraphPad Prism version 7.0. Genotype and allele frequencies were calculated within each group. Hardy–Weinberg equilibrium (HWE) was estimated online (https://www.oege.org/software/hwe-mr-calc.shtml) and tested by the goodness of fit. Overall comparison and subgroup analyses were performed. Adjusted odds ratios (OR) with a 95% confidence interval (CI) was calculated to identify the strength of the association between the SNP and cancer risk under various genetic association models^48^; allelic model (G *versus* A), homozygote comparison (GG *versus* AA), heterozygote comparison (AG *versus* AA), dominant model (GG + AG *versus* AA), and recessive model (GG *versus* AG + AA). The Wilcoxon matched-pair signed-rank test was carried out to compare the expression level between cancer samples and their corresponding adjacent non-cancer tissues. Chi-square (^2^) and Fisher's exact tests were used for qualitative parameters, while quantitative variables were shown as mean ± standard deviation (SD) or median (quartiles) according to data distribution. Spearman's correlation test was applied for correlation analysis. Overall survival time was counted (months) from the date of diagnosis to the date of death or last follow-up before study finalization. The Kaplan–Meier method and the Cox proportional hazard model were carried out to assess survival rates among groups. A two-tailed *P*-value < 0.05 was considered significant.

### Ethical approval

This study had been approved by the local Research & Ethics Committee, NBU, Arar, Saudi Arabia. The informed consent from the patients was waived from the ethical committee as the authors worked on archived FFPE samples.

## Supplementary information

Supplementary Figure 1.

## Data Availability

All data generated or analyzed during this study are included in this published article (and its Supplementary Information files).
