# Supplementary material for: Analysis of microRNA-34a expression profile and rs2666433 variant in colorectal cancer: a pilot study
Source: Sci Rep. 2020 Oct 9;10:16940. doi: 10.1038/s41598-020-73951-y (PMC7547073; doi:10.1038/s41598-020-73951-y)
Supplement: Supplementary file 1 — Supplementary Figure 1. [file 41598_2020_73951_MOESM1_ESM.pdf]

Analysis of microRNA-34a expression profile and rs2666433 variant in colorectal cancer- a pilot study

Manal S. Fawzy[1,6]*, Afaf T Ibrahiem[2,7] Baraah T. Abu AlSel[3], Saleh A. Alghamdi[4], Eman A. Toraih[5,8]

[1] *Department of Biochemistry, Faculty of Medicine, Northern Border University, Arar, Saudi Arabia*

[2] *Department of Pathology, Faculty of Medicine, Northern Border University, Arar, Saudi Arabia*

[3] *Department of Microbiology, Faculty of Medicine, Northern Border University, Arar, Saudi Arabia*

[4] *Medical Genetics, Clinical Laboratory Department, College of Applied Medical Sciences, Taif University, Taif, Saudi Arabia*

[5] *Department of Surgery, Tulane University, School of Medicine, New Orleans, Louisiana, USA*

[6] *Department of Medical Biochemistry and Molecular Biology, Faculty of Medicine, Suez Canal University, Ismailia, Egypt*

[7] *Department of Pathology, Faculty of Medicine, Mansoura University, Mansoura, Egypt*

[8] *Genetics Unit, Department of Histology and Cell Biology, Faculty of Medicine, Suez Canal University, Ismailia, Egypt*

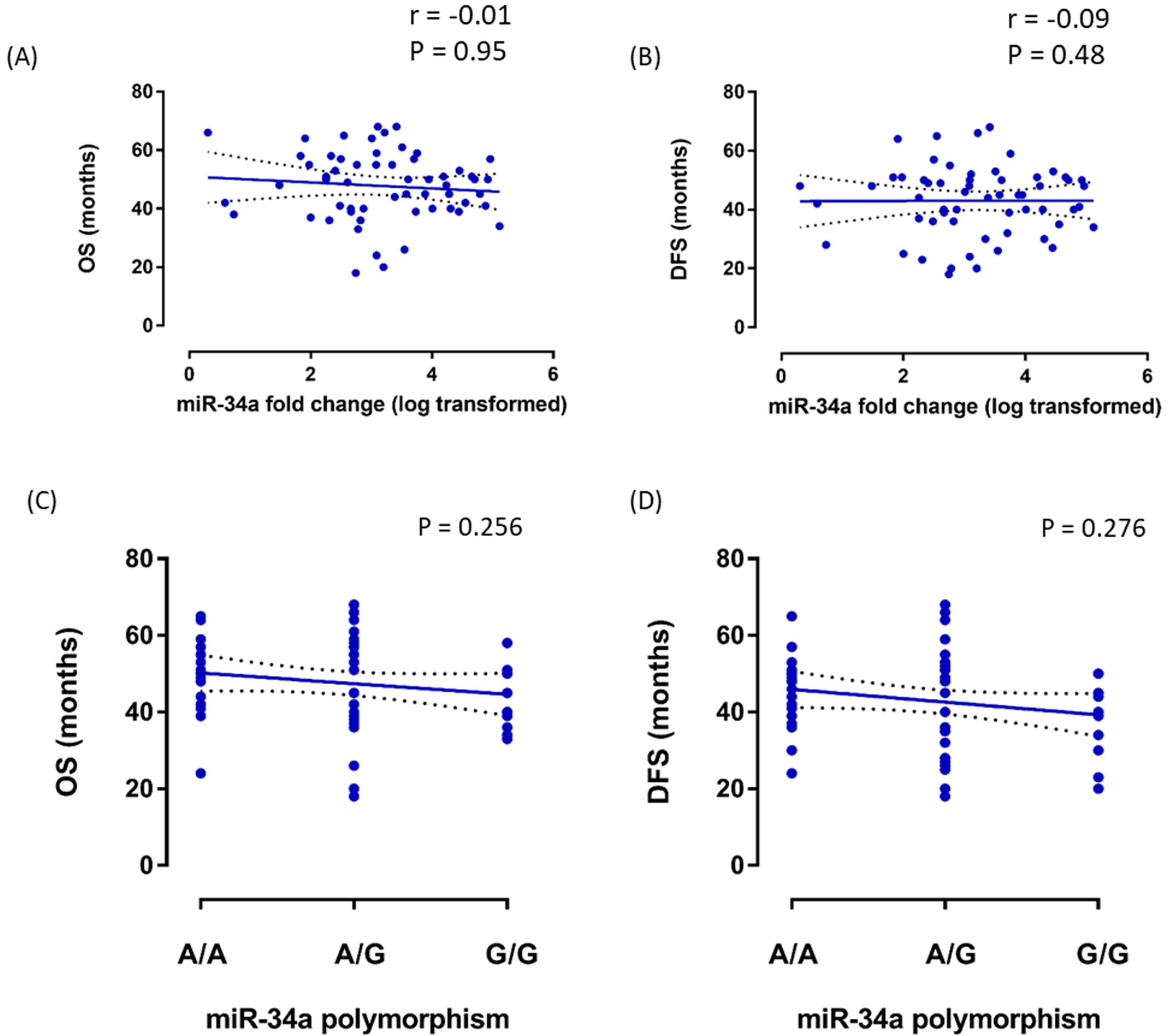

r = -0.01
P = 0.95
(A)
OS (months)
miR-34a fold change (log transformed)
r = -0.09
P = 0.48
(B)
DFS (months)
miR-34a fold change (log transformed)
(C)
P = 0.256
OS (months)
A/A
A/G
G/G
miR-34a polymorphism
(D)
P = 0.276
DFS (months)
A/A
A/G
G/G
miR-34a polymorphism

Figure S1. Correlation analysis between miR-34a and survival. (A) Expression level with overall survival (OS). (B) Expression level with disease-free survival (DFS). Expression values were log-transformed as data were non-parametric. Fold change was normalized to RNU6B and calculated using the delta-delta quantitative cycle method [=$2^{(-\Delta\Delta Cq)}$] in comparison to non-cancer adjacent tissues (C) rs2666433 polymorphism stratified by different genotypes with overall survival. (D) The rs2666433 polymorphism stratified by different genotypes with disease-free survival.
